# Evaluating the pH of Various Commercially Available Beverages in Pakistan: Impact of Highly Acidic Beverages on the Surface Hardness and Weight Loss of Human Teeth

**DOI:** 10.3390/biomimetics7030102

**Published:** 2022-07-26

**Authors:** Naresh Kumar, Faiza Amin, Danya Hashem, Sara Khan, Huma Zaidi, Sehrish Rahman, Tooba Farhan, Syed Junaid Mahmood, Muhammad Asif Asghar, Muhammad Sohail Zafar

**Affiliations:** 1 Department of Science of Dental Materials, Dr. Ishrat Ul Ebad Khan Institute of Oral Health Sciences, Dow University of Health Sciences, Karachi 74200, Pakistan; khansara4924@yahoo.com (S.K.); huma.zehra.zaidi@gmail.com (H.Z.); dr.sehrish.rahman@gmail.com (S.R.); toobafarhan20@hotmail.com (T.F.); 2 Department of Science of Dental Materials, Dow Dental College, Dow University of Health Sciences, Karachi 74200, Pakistan; faiza.ameen@duhs.edu.pk; 3 Department of Restorative Dentistry, College of Dentistry, Taibah University, Al Madinah, Al Munawwarah 41311, Saudi Arabia; dhashem@taibahu.edu.sa (D.H.); MZAFAR@taibahu.edu.sa (M.S.Z.); 4 Plastic and Polymer Section, Applied Chemistry Research Centre, PCSIR Laboratories Complex, Karachi 75280, Pakistan; junaid070880@gmail.com; 5 Food and Feed Safety Laboratory, Food and Marine Resources Research Centre, PCSIR Laboratories Complex, Karachi 75280, Pakistan; masif345@yahoo.com; 6 Department of Dental Materials, Islamic International Dental College, Riphah International University, Islamabad 44000, Pakistan

**Keywords:** beverages, pH, dental erosion, surface hardness, weight loss

## Abstract

The objectives of this study were to investigate the pH of common beverages and to evaluate the effects of common acidic beverages on the surface hardness and weight loss of human tooth specimens. A total of 106 beverages were conveniently purchased from supermarkets in Karachi, Pakistan. Prior to evaluation, beverages were refrigerated or stored at room temperature in accordance with the manufacturers’ recommendations. Beverages were categorized into six groups: ‘Sports and Energy drinks’, ‘Water’, ‘Fruit Juices and Drinks’, ‘Sodas’, ‘Milk and Flavored Milk’ and ‘Teas and Coffee’. Using a pH meter, the pH of each beverage was measured in triplicate at room temperature. In addition, the influence of five highly acidic beverages on the weight loss and surface hardness of human tooth specimens was evaluated using gravimetric analysis and the Vickers hardness tester, respectively. ‘Sports and Energy drinks’, ‘Fruits Juices and Drinks’ and ‘Sodas’ were the most acidic beverage categories, with a pH range of 3.00–5.00. A total of 33% of beverages tested in this study were highly acidic (pH less than 4.00), 29% of beverages were moderately acidic (pH 4.00–4.99) and 31% were mildly acidic (pH 5.00–6.99). Significant weight loss was observed in all immersed specimens compared to control counterparts (*p* < 0.05). Similarly, for surface hardness, five highly acidic beverages (Red Bull, Pepsi, Apple Cidra, Tang Mosambi and Tang Orange) significantly decreased the surface hardness of specimens (*p* < 0.05). The pH levels of commonly available beverages in Pakistan are highly acidic, which may encourage loss of minerals from teeth; hence, affecting their surface hardness.

## 1. Introduction

Dental erosion is a localized, chronic, painless loss of tooth enamel and dentine that have been chemically etched away from the surface of a tooth [1]. Globally, dental erosion affects 20% to 45% of permanent and 30% to 50% of deciduous dentitions [2]. Dental erosion is multifactorial and occurs due to consistent exposure to acidic fluids without microbial involvement [3]. The presence of hydrogen ions interrelates proton-promoted dissolution of fluorapatite and hydroxyapatite crystals present in tooth enamel and dentine [3]. Dental erosion can be classified into two major types: extrinsic, due to exposure to acidic foods and beverages and certain medicines, and intrinsic, as a result of gastroesophageal reflux disease and vomiting [4,5].

A major cause of extrinsic dental erosion is the consumption of acidic foods and beverages [6]. Sugary and acidic beverages have cariogenic and acidogenic tendencies, causing erosion of enamel and dental caries [7]. Additionally, in patients with gastrointestinal disorders such as gastroesophageal reflux disease (GERD), gastric acid from the stomach [8] may lead to the loss of tooth structure, hypersensitivity and compromised esthetics.

In modern society, youth are more attracted to the consumption of carbonated drinks. A major dilemma of the modern fast-track lifestyle is the increased intake of readily available carbonated drinks and juices. Consumption of fruit juices has been popularized as a healthy alternative to other beverages; this is a common modern myth and is the reason many parents give their children commercially available fruit juices [9]. In a study conducted by de Almeida et al. [9], researchers analyzed commercial fruit juices available in Brazil; they concluded that these fruit juices have low pH levels and high sugar contents [10].

There is a positive relationship between intake of acidic beverages and dental erosion. The severity of dental erosion can be affected by multiple factors including the duration, frequency, time of exposure and temperature of the beverages [11,12,13,14,15,16]. Enamel dissolution occurs at a critical pH of 5.5; however, loss of minerals may start at an even higher pH [17,18,19]. A study by Bello et al. concluded that intake of juices and soft drinks among residents accounted for 51% of total fluid intake [20]. In addition, dental erosion poses a more serious problem among athletes. Over 35% of university athletes have suffered from dental erosion [21,22]. A National Dental Health Survey conducted by Dugmore et al. [23] reported that 59.7% of 12-year-old British children suffered dental erosion. El Aidi et al. [24] reported that the prevalence of tooth erosion among 12-year-old school-going children was 32.2%, which increased to 42.8%. Data regarding dental erosion in Pakistan are very scarce. In one study, researchers evaluated 12–14 year-old school-going children and reported an association between dietary habits and dental erosion. The major etiological factors in the occurrence of dental erosion included the pattern and frequency of consuming acidic beverages [25]. It is alarming that more than 80% of the highly educated representative group of the Pakistani population consume such beverages [26].

For assessing dental erosion, different examination standards and scoring systems are reported in the literature. Therefore, it is often difficult to compare the outcomes of various epidemiological studies [27]. With this perspective, the present study incorporated the most commonly used method for categorization of beverages as follows: extremely erosive drinks (pH = 2.00–3.00), erosive (pH = 3.00–4.00) and minimally erosive (pH = 4.00–6.00) [28]. Considering the erosive potential of various beverages, the aims of this study were to thoroughly examine the pH levels of the most commonly consumed beverages in Pakistan and to evaluate their effects on the weight loss and surface hardness of human teeth.

## 2. Materials and Methods

This study was conducted after obtaining ethical approval from the institutional review board (IRB) of the Dow University of Health Sciences, Karachi, Pakistan (IRB-1648/DUHS/Approval/27 June 2020).

### 2.1. Evaluation of pH of Beverages

A total of 106 conveniently available beverages were purchased from local supermarkets in Karachi, Pakistan. Beverages were stored at 27 ± 1 °C prior to evaluation. Tea and coffee products (*n* = 6) were prepared by adding 10 g of each powder to 100 mL of boiling water which was then cooled to room temperature. Powdered drinks (*n* = 3) were prepared according to the manufacturer’s instructions noted on the packaging. ‘Ready to drink’ beverages (*n* = 96) were shaken well prior to opening.

**Figure 1:**
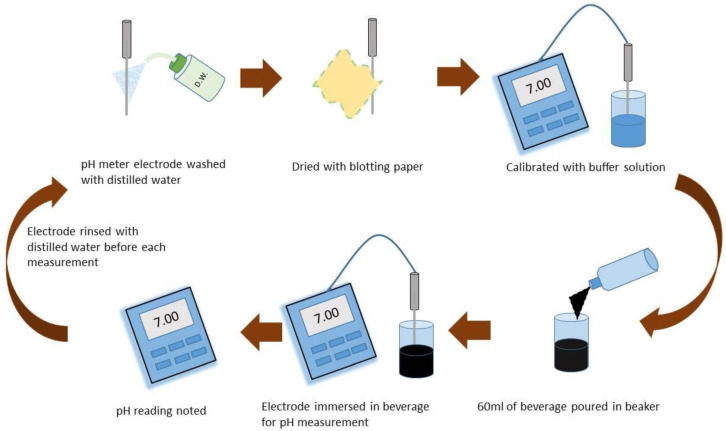
Schematic representation showing the procedure for pH measurement.

The pH of all beverages was determined using a pH meter (Model 720A, Thermoelectron Corp, Waltham, MA, USA). Measurements were taken immediately after removing the cork. The pH meter electrode was washed using distilled water and calibrated with HCL and NaOH as buffering solutions. Two beakers, ‘A’ and ‘B’, were filled with 100 mL distilled water and 60 mL of the experimental beverage, respectively. The electrode was first stirred in beaker A containing distilled water (pH 7.00). The pH electrode was then stirred in beaker ‘B’, containing an experimental beverage, and held stationary without touching the base or walls until stable readings were obtained on the pH meter. Three consecutive readings were recorded for each beverage. After each beverage testing, the beakers and the pH electrode were rinsed thoroughly with distilled water and dried with blotting paper before using them to test the next beverage (Figure 1).

Based on pH measurements, five highly acidic beverages (L1 = Red Bull, L2 = Pepsi, L3 = Apple Cidra, L4 = Tang Mosambi and L5 = Tang Orange) were selected for further evaluation regarding their effects on tooth structure loss and surface hardness.

### 2.2. Specimen Preparation

Five premolars extracted for orthodontic purposes were collected from the Department of Oral Surgery, Dow University of Health Sciences (DUHS), Karachi, Pakistan. Informed consent was obtained from patients prior to using their extracted human teeth. The teeth were washed and disinfected using 0.5% chloramine T trihydrate solution (Permata Scientific, Sendirian Berhad, Johor Bahru, Malaysia). The teeth were then scaled using a dental scaler (Peizon Master 400,EMS, Nyon, Switzerland) to remove any calculus and debris and were carefully examined to rule out the presence of any caries, enamel hypoplasia, stains, restorations, cracks or other defects. Teeth with any pathological conditions were excluded.

**Figure 2:**
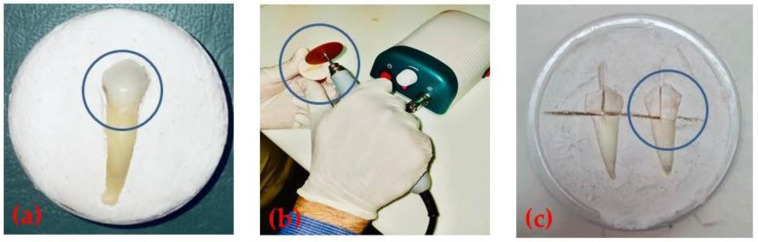
(**a**) Dried disinfected tooth. (**b**) Sectioning longitudinally using a microtome. (**c**) Cut into two longitudinal halves.

The cleaned and disinfected teeth were dried (Figure 2a) and longitudinally sectioned into two sections using a diamond cutting disc (MANI devices and instruments, Takenzawa, Japan) in a Micromotor (Figure 2b,c) (K-35 Cube 40,000 rpm, Seyang Micro Tech Co, Daegu, Korea). Each tooth section was labelled as follows: (A1, A2), (B1, B2), (C1, C2), (D1, D2) and (E1, E2). Subsequently, all sets of specimens were stored in distilled water until further experimentation. The thickness of each specimen was approximately 3 mm. Prior to testing, specimens were polished to remove debris, plaque and foreign particles. Briefly, samples were sequentially manually polished with silicon carbide papers of 600, 1200, 2500 and 4000 grit [29,30]. This was homogenous for all specimens. Moreover, the enamel surface of each specimen was exposed while the remaining part of the specimen was covered with nail varnish to simulate the oral environment.

### 2.3. Weight Analysis of Specimens

**Figure 3:**
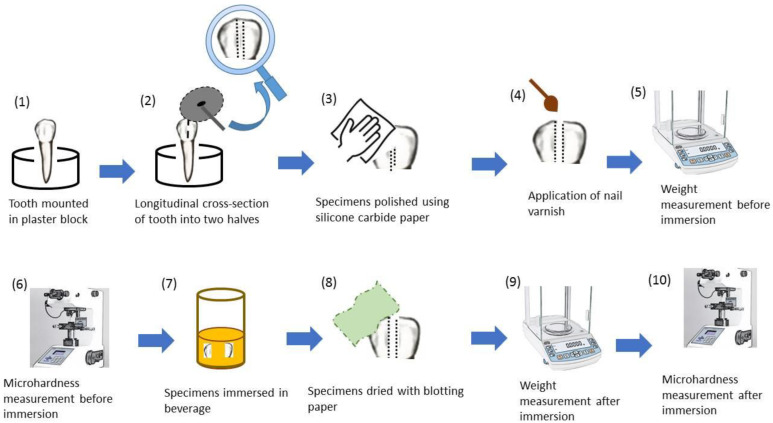
A schematic diagram showing a complete representation of the experiment from tooth sectioning to measurement of surface hardness and weight.

From each set, one specimen (A1, B1, C1, D1 and E1) was weighed for baseline readings before immersion into beverages. The specimens were dried with blotting papers for one hour at room temperature prior to recording the dry weight using an analytical balance (AL204 METTLER TOLEDO, Canada, accuracy 0.1 mg). The specimens (A1, B1, C1, D1 and E1) were then transferred to Falcon tubes (50 mL Conical Centrifuge Tubes, Fisher Scientific Co., Pittsburgh, PA, USA) containing 32.5 mL of a freshly opened beverage and labelled according to the name of the beverage (L1 to L5, respectively). Beverages were replaced every day until further analysis after the completion of a 7-day immersion period (Figure 3).

### 2.4. Surface Hardness Testing

Surface hardness testing of specimens (A2, B2, C2, D2 and E2) was performed using the Vickers tester (ZHV Hardness Tester, ZwickRoell Indentec, Brierley Hill UK) at 100*g* load with a loading time of 15 s. Three indentations were performed for each specimen, which were then placed in beverages L1 to L5, respectively. After the 7-day immersion period, specimens were rinsed with distilled water, dried with blotting paper for one hour and analyzed using the surface hardness test (Figure 3).

### 2.5. Data Analysis

The mean and standard deviations for pH values of each beverage were recorded. One-way analysis of variance (ANOVA), including post hoc Tukey’s test, was conducted on the surface hardness and weight data to assess the difference between pre- and post- immersion tooth specimens.

## 3. Results

Twenty-one (*n* = 21) sports and energy drinks had pH values in the range of 3.04 (Holsten Black Grapes flavor) to 4.58 (Three Horses), with a mean and standard deviation of 3.81 and (0.00), respectively (Table 1). Water from five different companies (*n* = 5) had a pH range of 7.0 (Aquafina) to 7.63 (Nestle Pure Life), the mean and standard deviation of this group was 7.32 and (0.00), respectively (Table 2). Fruit juices and drinks (*n* = 33) had a pH range of 3.15 (Tang Mosambi) to 5.22 (Nestle Fruita Vitals Royal Mangoes), with a mean and standard deviation of 4.22 and (0.00), respectively (Table 3). Sodas (*n* = 16) had a pH range of 3.40 (Pepsi) to 4.59 (Pakola Cream Soda), with a mean and standard deviation of 3.77 and (0.00), respectively (Table 4). The average mean and standard deviation of the tea and coffee group was 5.99 and (0.01), respectively, with a pH range of 5.08 (Lipton Tea Yellow Label) to 6.88 (Nescafe Chilled Mocha) (Table 5). Milk products (*n* = 23) had a pH range of 6.27 (Go Fresh Coconut Milk Drink Plus Coconut Water Nata De CoCo Rose Flavor) to 7.87 (Nestle Milo), with a mean and standard deviation of 6.45 and (0.00), respectively (Table 6).

Out of 106 drinks, 35 (33%) were found to be erosive with a pH value in the range of 3.00–3.99, and 31 (29%) beverages were minimally erosive (pH = 4.00–4.99). Similarly, 33 beverages were considered minimally erosive with pH values in the range of 5.00–6.99. None of the tested beverages were highly erosive. Water from all companies demonstrated a neutral pH range of 7.00–7.20, and five drinks had a pH range of 7.20–7.87.

In terms of weight analysis, significant weight loss was observed in all immersed specimens compared to control counterparts (*p* < 0.05) (Table 7). Similarly, for surface hardness, all five highly acidic beverages, namely Red Bull, Pepsi, Apple Cidra, Tang Mosambi and Tang Orange, significantly decreased the surface hardness of specimens (*p* < 0.05) (Table 8).

## 4. Discussion

The present study investigated pH levels of a wide variety of commonly used beverages. In addition, the erosive effects of various acidic beverages on dental hard tissues were determined by evaluating variations in the surface hardness and loss of minerals from human tooth specimens. The present study is the first of its kind to investigate a wide variety of beverages, including bottled waters, sports/energy drinks, fruit juices, carbonated sodas, flavored milks, coffees and teas; hence, it presents comprehensive and diverse data regarding the effects of these beverages on dental tissues. In contrast, previous studies [31,32] determined the pH of a limited number (maximum 14) of beverages and their effect on tooth erosion. Most of the beverages investigated in this study had an acidic pH. Highly acidic beverages significantly affected the weight loss and surface hardness of human tooth specimens (*p* < 0.05).

For the evaluation of beverages’ erosive potential, pH is considered the most important key factor [33,34]. In this study, we utilized an inverse logarithmic relationship reported by Larsen and Nyvad [28] to determine beverages’ pH and erosive potential. This method has also been employed by other researchers [35]. Beverage manufacturing companies do not usually print pH information on their product labels. Therefore, the present study, together with similar previous studies, provide invaluable data to health care workers and the general population.

Although the pH and resultant erosive potential of beverages have widely been reported in previous studies [21,33,35,36], it is difficult to compare these data with beverages available in Pakistan due to gross variations in manufacturing processes, temperature and equipment accuracy. For instance, beverages tested at a higher temperature exhibited lower pH values [37].

In terms of pH data, results in this study are not in agreement with previous studies conducted in Pakistan. For instance, Haq et al. [31] reported the pH values of Pepsi and Mountain Dew as 2.60 and 2.94, respectively; however, in this study the pH values for both drinks were observed as 3.47 and 3.87, respectively. This difference might be attributed to different scoring systems used in these studies. Similarly, in a previous study a sports drink (‘Sting’) exhibited a pH value of 3.0 [32]; in contrast, this study reported a pH value of 4.80 for the same drink. This variation in findings may be attributed to different methodologies and distinct pH testing methods. In this study, a pH electrode meter was used to evaluate the pH of various beverages with three consecutive readings; whereas their study used pH strip methods and performed experiments for 15 days. Four cycles were conducted each day at six-hour intervals.

This study clearly indicates that many of the commercially available, non-alcoholic beverages in Pakistan have the potential to cause dental erosion (Table 1, Table 2, Table 3, Table 4, Table 5 and Table 6). The increase in consumption of these beverages highlights a potentially serious oral and general health risk. Awareness of beverage pH is critical for designing preventive policies for patients susceptible to clinical erosion [38,39,40]. Minimization of erosive drinks (pH 3.00–3.99) and replacement with drinks having a pH of 4.00 or higher would be sensible recommendations for the prevention of erosion.

These data may be used as a reference for future research and may also provide health experts and individuals with an instant resource when advocating a healthy diet to and for consumers. Undoubtedly, these data regarding pH findings are significant and alarming for the health of human dentition.

The common methods used to analyze tooth erosion include surface hardness, scan electron microscopy, microradiography, chemical analysis, digital image analysis and atomic force microscopy [41]. In this study, we chose surface hardness (considered an effective approach to measure the change in a tooth’s surface microstructure), which is indirectly suggestive of the degree of demineralization. The findings of this study related to surface hardness clearly highlight the significant effect of all five acidic beverages on tooth structure (Table 8), which is in agreement with a previous study [42]. Jeong M.J et al. [42] evaluated the effect of four energy drinks, including ‘Red Bull’, on the surface hardness of tooth specimens; they observed a significant difference between the surface hardness values of pre- and post-immersion specimens.

The surface hardness of the Tang Orange treated specimen decreased more significantly compared to the other four tested beverages, although the pH values are not significantly different. This finding may be due to the presence of different ingredients in Tang Orange. It is a well-known fact that ingredients play a key role in dental erosion. For instance, the presence of citric acid and sugars in beverages has been considered a significant cause of dental erosion. Further studies with regard to the precise composition of beverages and scan electronic microscopic examination of beverage-treated specimens are warranted so as to discover the actual cause of such a finding [43,44].

There were significant differences among the hardness values of specimens. Since the specimens in our study were made from teeth of different patients, differences in the orientation of enamel rods, the orientation of the crystallites within the rods, the degree of demineralization and the presence of fluoride ions are expected. This might have led to distinct hardness values among the specimens evaluated in the study.

Weight loss of tooth structure is also considered a suitable method to predict the effect of beverages on erosion. Methew et al. [45] evaluated the effect of seven beverages (Pepsi, Red Bull, orange juice, apple juice, lemon juice, coffee and green tea) on the human tooth over a one-month period. They observed a significant loss of tooth structure with orange juice, Red Bull and Pepsi, which is in agreement with this study, as substantial tooth loss was evident with all five acidic beverages in our findings (Table 7). In another study, Bitri et al. [46] conducted an in vitro analysis to assess the erosive effect of some common drinks. The authors employed the weight-loss method and identified erosive effects in terms of dental hard tissue dissolution with all soft drinks evaluated. Weight loss in both of these studies cannot be compared, owing to methodological differences.

There are a few limitations in the present in vitro study. The buffering capacity and flushing action of saliva, which may potentially influence the erosive action of acidic beverages, was not simulated. In addition, morphological assessment to analyze the materials’ surface variations following acidic exposition were not investigated. Additionally, due to cultural and religious constraints, alcoholic beverages were not evaluated in this study.

Accordingly, despite the significant clinical relevance of this study, further research is essential to investigate erosion kinetics, surface corrosion, surface morphological analysis and diffusion in the solid state.

## 5. Conclusions

Under the limitations of this study, it was concluded that out of 106 beverages, the pH levels of 99 drinks in Pakistan were determined to be acidic, and hence, considered erosive. Moreover, five acidic beverages, namely Red Bull, Pepsi, Apple Cidra, Tang Mosambi and Tang Orange, clearly demonstrated erosive effects through the decreased surface hardness and weight loss of human tooth specimens.

## Figures and Tables

**Table 1:** pH (mean and standard deviation) of sports drinks/energy drinks.

S.No.	Sports Drinks/Energy Drinks	pH (Standard Deviation)	Batch No.
**Erosive**			
1	Holsten Black Grapes Flavor	3.04 (0.00)	132A13
2	Bavaria Holland Peach	3.18 (0.00)	CLFB40612R
3	Bavaria Holland Pomegranate	3.22 (0.00)	CLFB40612R
4	Bavaria Holland Mango Passion	3.25 (0.00)	CLFB40612R
5	Bavaria Holland Strawberry	3.30 (0.00)	CLFB 406 12R
6	Bavaria Holland Apple	3.31 (0.00)	CLFB 406 12R
7	Walkers’ Ginger Beverage	3.33 (0.00)	**
8	Red Bull	3.65 (0.00)	8L01B07C
9	Activade Grapes	3.83 (0.00)	ACT34
10	Gatrorade Blue Bolt	3.98 (0.01)	14:20 P 071120
**Minimally Erosive**			
11	** Malt Beverage Barbican	4.01(0.00)	**
12	Activade Berry Blue	4.06 (0.00)	ACT32
13	Activade Lemon Lime	4.07 (0.00)	ACT31
14	Gatrorade Tropical Fruit	4.09 (0.00)	
15	Activade Orange	4.14 (0.00)	
16	Gatrorade White Lightning	4.15 (0.00)	B 13:16 P 081020
17	Activade Fruit Punch	4.15 (0.00)	ACT33
18	Sting Energy Berry Blast	4.17 (0.00)	P281120G58
19	Sting Gold Rush	4.20 (0.00)	P101120638
20	**Malt 79 Murree Brewery	4.42 (0.00)	33H1 09:59
21	Three Horses	4.58 (0.00)	MRP17202

** Batch no. of beverage is not provided by the manufacturer.

**Table 2:** pH (mean and standard deviation) of waters.

pH of Waters			
	Waters	pH (Standard Deviation)	Batch No.
	**Non Erosive**		
22	Aquafina	7.0 (0.00)	15:46
23	Masafi Water	7.1 (0.00)	**
24	Mai Dubai	7.33 (0.00)	**
25	Nestle Pure Life Active Water	7.58 (0.00)	010215801A
26	Nestle Pure Life	7.63 (0.00)	028330621D

** Batch no. of beverage is not provided by the manufacturer.

**Table 3:** pH (mean and standard deviation) of fruit juices and drinks.

pH of Fruit Juices and Drinks			
	Fruit Juices/Drinks	pH (Standard Deviation)	Batch No.
**Erosive**			
27	Tang Mosambi	3.15 (0.01)	OTG 6301341
28	Tang Orange Flavor	3.16 (0.01)	
29	Tang Lemon and Pepper	3.19 (0.00)	
30	Smart Choice Pineapple with Pulp	3.49 (0.00)	**
31	Lemonade (Active Foods) Mint Lemonade	3.52 (0.00)	LIM 33
32	Limonade (Active Foods)	3.56 (0.00)	LIM02
33	Smart Choice Peach with Pulp	3.66 (0.00)	**
34	Smart Choice Apple with Pulp Drink	3.70 (0.00)	**
35	Fruiti-O Guava Nectar	3.91 (0.00)	21004376L2
36	Smile Lychee Flavor	3.94 (0.00)	21091291 L2
37	Must Mango Fruit Drink	3.96 (0.00)	1008(16:02:07)
38	Fruiti-O Peach Fruit Drink	4.00 (0.00)	20031102009735368001 L2
**Minimally Erosive**			
39	Smile Apple	4.04 (0.00)	**
40	Smart Choice Red Grape	4.12 (0.00)	**
41	Fruitien Red Grapes Fruit Drink	4.18 (0.00)	20075:6L3E
42	Hemani Peach Drink with Basil Seeds	4.20 (0.00)	EX007R2309
43	Nestle Fruita Vitals Red Grapes	4.24 (0.00)	028015801L
44	Slice Mango Fruit Drink	4.24 (0.00)	PX12B21:53
45	Fruitien Pomegranate Nectar	4.29 (0.00)	0024826L
46	Nestle Fruita Vitals Pineapple	4.50 (0.00)	2.5E + 08
47	Fruitien Joy Mango Fruit Drink	4.52 (0.00)	202892132L4
48	Fruitien Pineapple Nectar	4.54 (0.00)	**
49	Hemani Cocktail Drink with Basil Seeds	4.57 (0.00)	EX007R0103
50	Nestle Fruita Vitals Peach Fruit Drink	4.60 (0.00)	018215801Z
51	Hemani Lychee Drink with Basil Seeds	4.63 (0.00)	EX007R1202
52	Anytime Green Apple Fruit Nectar	4.66 (0.00)	124(05:4248)
53	Hemani White Grapes with Basil Seeds	4.77 (0.00)	**
54	Nestle Fruita Vitals Apple Nectar	4.82 (0.00)	0309158010(13:59)
55	Nestle Fruita Vitals Kinnow Nectar	4.95(0.00)	031715803H(05:21)
56	Fruitien Chaunsa Mango Nectar	5.02 (0.00)	20276411L3E
57	Nestle Fruita Vitals Chaunsa Mango Nectar	5.03 (0.00)	031515802G
58	Nestle Fruita Vitals Guava Nectar	5.10 (0.00)	2.5E + 08
59	Nestle Fruita Vitals Royal Mangoes	5.22 (0.00)	3.1E + 08

** Batch no. of beverage is not provided by the manufacturer.

**Table 4:** pH (mean and standard deviation) of sodas.

pH of Sodas			
	Soda	pH (Standard Deviation)	Batch No.
**Erosive**			
60	Pepsi	3.40 (0.00)	P301120638 04:17
61	Forest Club Soda	3.47 (0.00)	**
62	Pepsi Diet	3.52 (0.00)	P061120GA 03:46
63	Coca Cola Original Taste	3.54 (0.00)	1319L3
64	Pakola (Lychee)	3.55 (0.00)	**
65	Pakola Lemon Lime	3.61 (0.00)	22106
66	Pepsi Cola	3.62 (0.00)	
67	Apple Sidra	3.73 (0.00)	
68	Mirinda Orange Flavor	3.78 (0.00)	P241120638
69	Fanta Orange Flavor	3.84 (0.00)	0205M6PP36
70	Mountain Dew	3.87 (0.00)	P281020058
71	Vimto Sparkling	3.91 (0.01)	661JLY21
72	7Up	3.95 (0.00)	
73	Sprite	3.96 (0.00)	
**Minimally Erosive**			
74	7Up Free	4.06 (0.00)	P26102003A
75	Pakola Cream Soda	4.59 (0.00)	21APR217UMD

** Batch no. of beverage is not provided by the manufacturer.

**Table 5:** pH (mean and standard deviation) of teas and coffees.

	pH of Teas and Coffees		
	Teas and Coffees	pH (Standard Deviation)	Batch No.
**Minimally Erosive**			
76	Lipton Tea Yellow Label	5.08 (0.01)	5
77	Tea Supreme	5.11 (0.02)	5
78	Tapal Family Mixture	5.31 (0.00)	509234
79	Tapal Danedar	5.46 (0.00)	513252
80	Green Tea Lipton	6.65 (0.01)	
81	Nescafe Chilled Latte	6.70 (0.00)	3.1E + 08
82	Nescafe Coffee	6.73 (0.01)	
83	Nescafe Chilled Mocha	6.88 (0.00)	029715801d

** Batch no. of beverage is not provided by the manufacturer.

**Table 6:** pH (mean and standard deviation) of milks and flavored milks.

pH of Milks and Flavored Milks			
	Milks	pH (Standard Deviation)	Batch No.
**Minimally Erosive**			
84	Go Fresh Coconut Milk Drink plus Coconut Water with Nata De CoCo Rose Flavour	6.27 (0.00)	RA189 2205J1626
85	Go Fresh Coconut Milk Drink plus Coconut Water with Melon Flavor	6.29(0.00)	RA189 2205J1809
86	Day Fresh Milk Full Cream	6.51 (0.00)	0333P1B7
87	Nurpur Full Cream Milk	6.52 (0.00)	
88	Day Fresh Flavored Milk Banana	6.53 (0.00)	0186B1C4
89	Day Fresh Flavored Milk Strawberry	6.58 (0.00)	0294S1B6
90	Olpers Full Cream Milk	6.60 (0.00)	
91	Oolala Flavored Milk Strawberry Shakarganj	6.61 (0.00)	13B(13:36:12)
92	Olpers Chocolate Flavored Milk	6.65 (0.00)	20(13:38:59)
93	Tarang (liquid tea whitener)	6.65 (0.00)	173(04:28:38)
94	Nestle Milk Pak Full Cream	6.69 (0.00)	032315801C
95	Day Fresh Flavored Milk Mango	6.70 (0.00)	0256M1C7
96	Oolala Flavored Milk Badam and Zaffran	6.71(0.00)	125LFM(05:35:33)
97	Olpers Pro Cal Low Fat Milk	6.72 (0.00)	D0233(19:26:43)
98	Day Fresh Flavored Milk Pista Zaffran	6.73(0.00)	0292Z1b7
99	Nestle Nesvita	6.77 (0.00)	030915801U
100	Nurpur Flavored Milk Badam and Zaffran	6.84 (0.00)	32(06:41:56)
101	Pakola Chocolate Flavored Milk	6.90 (0.00)	GS 12:44 B05
102	Go Fresh Coconut Milk Drink with Chocolate	6.93 (0.00)	
103	Pakola Flavored Milk Strawberry	6.94 (0.00)	**
104	Oolala Chocolate Flavored Milk Shakarganj	6.97 (0.00)	011(12:29:05)
**Non Erosive**			
105	Pakola Double Delight Strawberry plus Vanilla	7.07 (0.00)	**
106	Milo Nestle	7.87 (0.00)	028515801U

** Batch no. of beverage is not provided by the manufacturer.

**Table 7:** Weight of specimens before and after immersion in highly acidic beverages (mean and SD).

Beverage Type	Red Bull	Pepsi	Apple Cidra	Tang Mosambi	Tang Orange
**Pre-immersion weight** **(grams)**	0.18 (±0.00)	0.08 (±0.00)	0.10 (±0.00)	0.12 (±0.00)	0.16 (±0.01)
**Post-immersion Weight** **(grams)**	0.11 (±0.00)	0.04 (±0.00)	0.05 (±0.00)	0.07 (±0.00)	0.11 (±0.00)
**Weight Reduction in Specimens After Immersion (%)**	38.89	50.00	50.00	41.67	31.25
***p* Value**	*p* = 0.000	*p* = 0.001	*p* = 0.000	*p* = 0.000	*p* = 0.003

*p* < 0.05 indicates a statistically significant difference between pre- and post-immersion specimens.

**Table 8:** Surface hardness of specimens before and after immersion in highly acidic beverages (mean and SD).

Beverages Type	Red Bull	Pepsi	Apple Cidra	Tang Mosambi	Tang Orange
**Pre-immersion Hardness** **(VHN)**	598 (±50)	553 (±13)	686 (±9)	669 (±17)	654 (±40)
**Post-immersion Hardness** **(VHN)**	473 (±20)	457 (±16)	606 (±21)	566 (±21)	347 (±46)
**Surface Hardness Reduction in Specimens After Immersion (%)**	21	17	12	15	47
***p* Value**	*p* = 0.001	*p* = 0.001	*p* = 0.003	*p* = 0.003	*p* = 0.001

*p* < 0.05 indicates a statistically significant difference between pre- and post-immersion specimens.

## Data Availability

The data presented in this study are available on the request from the corresponding author.
